# Supplementary material for: Chromothripsis during telomere crisis is independent of NHEJ, and consistent with a replicative origin
Source: Genome Res. 2019 May;29(5):737–49. doi: 10.1101/gr.240705.118 (PMC6499312; doi:10.1101/gr.240705.118)
Supplement: Supplemental Material [file supp_gr.240705.118_Supplemental_file_1.zip › contigs/annotated_contigs/DB83/contig.2.DB83_length_474_mean_cov_8.71097046414.docx]

**DB83_length_474_mean_cov_8.71097046414**

```
GGGCTATGTCTAACTGGCAAGATGAAGCTTCCCTGAGAAGATTGTGTCTGAATCTCACAGAAAAGAAATACGAATAAGGCAGTTTAGCA
 >chr17:16311573-16311813 - E=7e-124
GGCAGCACAGGCTTCCTGGAGCAAGGAAGACTGAGGCAGCGGAGAAAAAAAAATGCTAGCTGGTCCTCAAACAGGGCCAGAGAGGACCA

AAGGGGACCAGACACCTCCTGGAGGGCAAGGGGACCAGCTGGCTGGGCACAGCC|TGCCCA|CCACCACACCTGGCTAGTTTTTGAATT
                                                       >chr17:16309772-16309982 - E=8e-
TTTAGTAGAGACCAGATTCCACCATGTTGGCCAGGCTAATCTCGAACTCCTGACCTCAGTGATCTGCCGGCCTTGGCCTCTCAAAATGT
115
TGAGATTACAGGCGTGAGCCACCTCTGCAGGCCAACACTCTTTCTTTCTTTCTTTCTTTCTTTCTTTCTTTCTTTCTTTCTTTCTTTC|

TTTCCTTTTCTTTCTTTCCTTCTTTCTTTTTT
```
